# In a Prediabetic Model, Empagliflozin Improves Hepatic Lipid Metabolism Independently of Obesity and before Onset of Hyperglycemia

**DOI:** 10.3390/ijms222111513

**Published:** 2021-10-26

**Authors:** Martina Hüttl, Irena Markova, Denisa Miklankova, Iveta Zapletalova, Martin Poruba, Martin Haluzik, Ivana Vaněčkova, Hana Malinska

**Affiliations:** 1 Centre for Experimental Medicine, Institute for Clinical and Experimental Medicine, 14321 Prague, Czech Republic; martina.huttl@ikem.cz (M.H.); irena.markova@ikem.cz (I.M.); denisa.miklankova@ikem.cz (D.M.); 2 Department of Pharmacology, Faculty of Medicine and Dentistry, Palacky University Olomouc, 77900 Olomouc, Czech Republic; iveta.zapletalova@upol.cz (I.Z.); martin.poruba@upol.cz (M.P.); 3 Diabetes Centre, Institute for Clinical and Experimental Medicine, 14321 Prague, Czech Republic; martin.haluzik@ikem.cz; 4 Department of Experimental Hypertension, Institute of Physiology, Czech Academy of Sciences, 14220 Prague, Czech Republic; ivana.vaneckova@fgu.cas.cz

**Keywords:** SGLT-2 inhibitors, empagliflozin, fatty liver, lipid metabolism, cytochrome P450, fetuin-A, oxidative stress

## Abstract

Recent studies suggest that treatment with SGLT-2 inhibitors can reduce hepatic lipid storage and ameliorate non-alcoholic fatty liver disease (NAFLD) development beyond their glycemic benefits. However, the exact mechanism involved is still unclear. We investigated the hepatic metabolic effect of empagliflozin (10 mg/kg/day for eight weeks) on the development of NAFLD and its complications using HHTg rats as a non-obese prediabetic rat model. Empagliflozin treatment reduced neutral triacylglycerols and lipotoxic diacylglycerols in the liver and was accompanied by significant changes in relative mRNA expression of lipogenic enzymes (*Scd-1*, *Fas*) and transcription factors (*Srebp1*, *Pparγ*). In addition, alterations in the gene expression of cytochrome P450 proteins, particularly *Cyp2e1* and *Cyp4a*, together with increased *Nrf2,* contributed to the improvement of hepatic lipid metabolism after empagliflozin administration. Decreased circulating levels of fetuin-A improved lipid metabolism and attenuated insulin resistance in the liver and in peripheral tissues. Our results highlight the beneficial effect of empagliflozin on hepatic lipid metabolism and lipid accumulation independent of obesity, with the mechanisms understood to involve decreased lipogenesis, alterations in cytochrome P450 proteins, and decreased fetuin-A. These changes help to alleviate NAFLD symptoms in the early phase of the disease and before the onset of diabetes.

## 1. Introduction

Non-alcoholic fatty liver disease (NAFLD) is a hepatic manifestation of metabolic syndrome, obesity, and various prediabetic states and is characterized by increased hepatic lipid accumulation (˃5%). Considered an independent risk factor for cardiovascular events [1,2], NAFLD markedly increases the risk of developing type 2 diabetes mellitus (T2DM) [3]. Along with impaired lipid metabolism and ectopic lipid deposition, dyslipidemia is one of the most common disorders linked to prediabetic conditions and is often preceded by the onset of hyperglycemia.

Although approximately 50% of obese individuals and up to 70% of diabetic patients exhibit characteristic features of fatty liver, NAFLD can also occur in non-obese individuals. The prevalence of non-obese NAFLD ranges widely from 3 to 30% [4].

Despite the high prevalence of NAFLD, an effective therapy has yet to be developed. However, drugs with antidiabetic properties, such as thiazolidinediones, glucagon-like peptide-1 (GLP-1) analogs, and metformin, can attenuate the development and progression of NAFLD. Sodium-glucose cotransporter-2 (SGLT-2) inhibitors are a class of drugs that exert pleiotropic effects on NAFLD. One of the most promising is empagliflozin [5,6]. According to recent randomized clinical studies, empagliflozin treatment ameliorates hepatic fat accumulation in diabetic subjects [7,8], with one study even noting an effect in NAFLD patients without T2DM [9]. A recent meta-analysis on SGLT-2 treatment of NAFLD disease involving 850 overweight and obese individuals [10] found that SGLT-2 inhibitors significantly decreased liver enzymes and the absolute percentage of liver fat content, as assessed by magnetic resonance techniques. However, there was limited evidence of any beneficial effect on histological outcomes.

In animal studies, empagliflozin administration has been shown to decrease hepatic lipid accumulation and ameliorate signs of NAFLD in models of diet-induced obesity and diet-induced NAFLD followed by T2DM onset [11,12,13]. In addition, empagliflozin administration in high-fat-fed mice has been shown to alleviate hepatic insulin resistance and improve lipid metabolism in the liver [11,12]. Although the exact mechanisms are not yet understood, the results of animal and human studies [5,6] indicate that empagliflozin has pleiotropic metabolic effects, supplementing its recognized glycemic benefits. The results of studies involving obese mice with diet-induced NAFLD point to the beneficial effects of empagliflozin on hepatic inflammation and the endoplasmic reticulum stress pathway, helping to mitigate signs of hepatic steatosis [11,12]. 

However, the potential advantageous effects of empagliflozin on NAFLD have not yet been studied independently of obesity or before the onset of hyperglycemia. To address this deficit, our study investigated the metabolic effect of empagliflozin on hepatic lipid and glucose metabolism in hereditary hypertriglyceridemic (HHTg) rats, a non-obese prediabetic rat model [14,15]. These animals exhibit genetically determined hypertriglyceridemia, insulin resistance in peripheral tissues, and ectopic lipid accumulation in the absence of obesity and hyperglycemia. Therefore, we hypothesized whether empagliflozin treatment could ameliorate the development and progression of NAFLD in non-obese rat models with fatty liver.

## 2. Results

### 2.1. Characterization of Metabolic Parameters in HHTg Rats

Prior to empagliflozin administration, there were no differences in initial body weight, glucose or serum triacylglycerols (TAG) between the control Wistar and Wistar + empa groups or between the prediabetic HHTg and HHTg + empa groups. Empagliflozin administration slightly increased food intake (+7%, *p* ˂ 0.05) but had no effect on drink intake.

**Figure 1:**
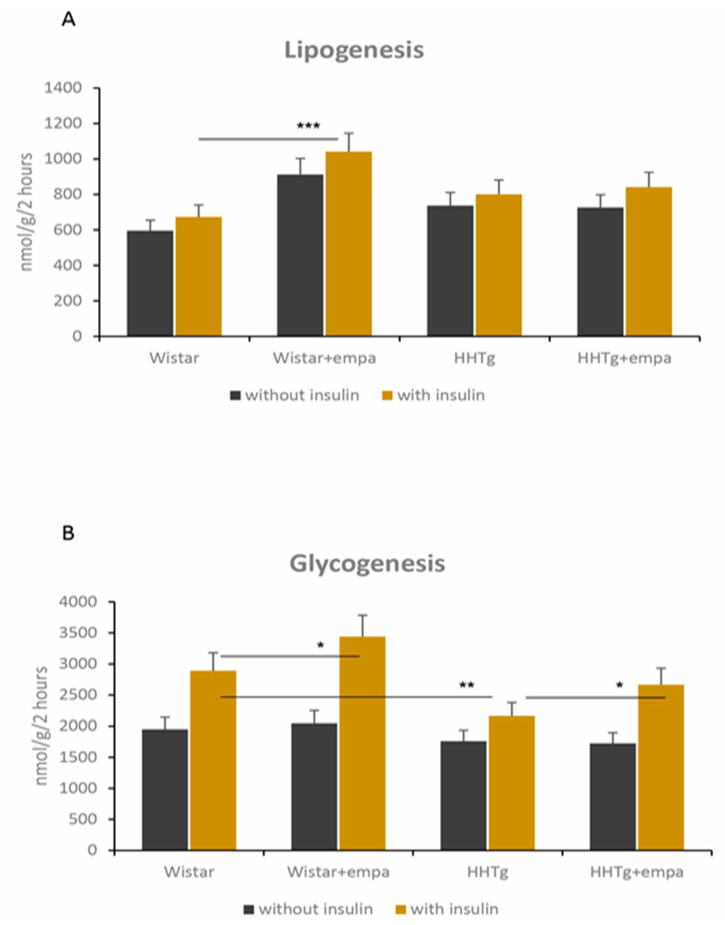
Effect of empagliflozin on skeletal muscle (glycogenesis) (**A**) and adipose tissue (lipogenesis) insulin sensitivity (**B**) in Wistar control and prediabetic HHTg rats. Data are expressed as mean ± SEM; n = 8 for each group; * denotes *p* ˂ 0.05, ** denotes *p* < 0.01, *** denotes *p* < 0.001.

**Figure 2:**
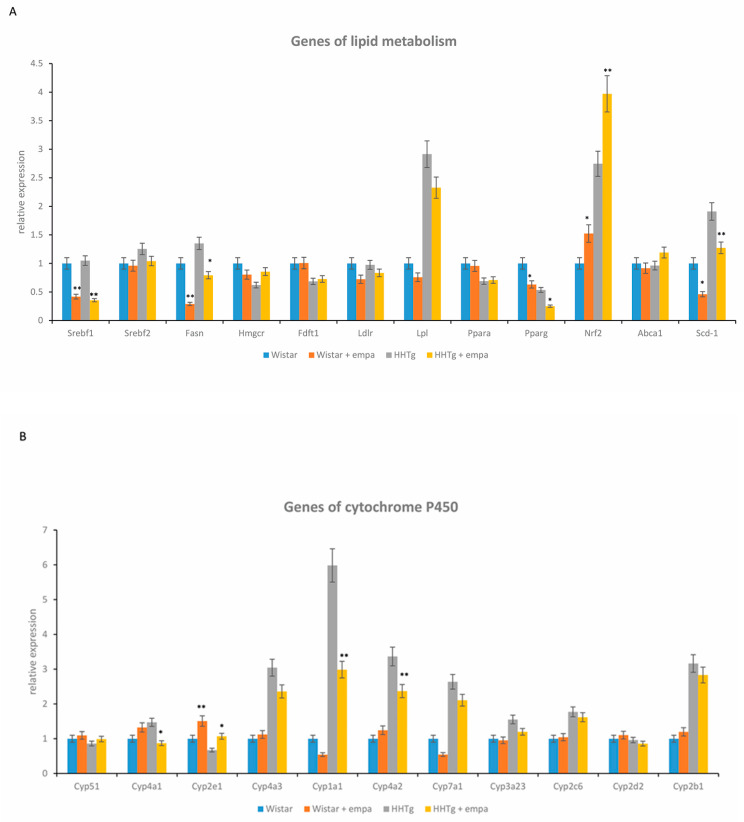
Effect of empagliflozin on hepatic gene expression of lipid metabolism (**A**) and cytochrome P450 family proteins (**B**) in Wistar control and prediabetic HHTg rats. Data are expressed as mean ± SEM; n = 8 for each group; * denotes *p* < 0.05, ** denotes *p* < 0.01. *Srebf*—sterol regulatory element-binding protein; *Fasn*—fatty acid synthase; *Hmgcr*—3-hydroxy-3-methylglutaryl-coenzyme A reductase; *Fdft*—farnesyl-diphosphate farnesyltransferase; *Ldlr*—LDL receptors; *Lpl*—lipoprotein lipase; *Pparα*—peroxisome proliferator-activated receptor alpha; *Pparg*—peroxisome proliferator-activated receptor gamma; *Nrf2*—nuclear factor erythroid-2-related factor 2; *Abca*—abc transporters; *Scd*—stearoyl-coenzyme A desaturase.

**Figure 3:**
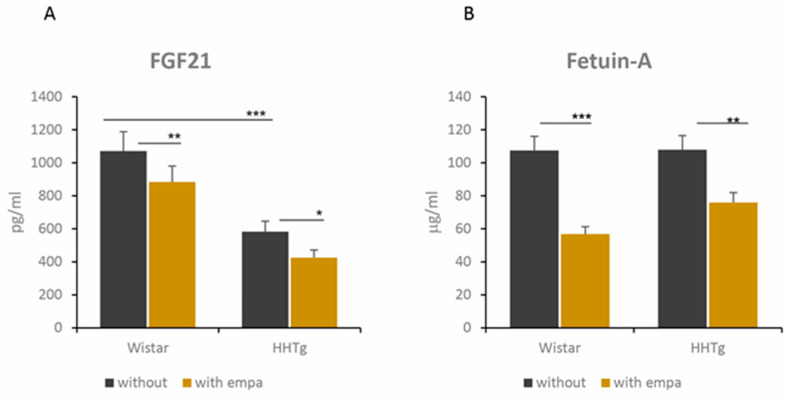
Effect of empagliflozin on circulating hepatokines levels in Wistar control and prediabetic HHTg rats—(**A**) FGF21, (**B**) Fetuin-A. Data are expressed as mean ± SEM; n = 8 for each group; * denotes *p* < 0.05, ** denotes *p* < 0.01, *** denotes *p* < 0.001.

Compared to Wistar controls, untreated HHTg animals exhibited markedly increased serum TAG and NEFA (Table 1). Hypertriglyceridemia in HHTg rats was associated with impaired glucose tolerance, represented by elevated non-fasting glucose and AUC. We also observed a significant increase in the pro-inflammatory markers leptin, MCP-1, TNFα, and IL-6 in untreated HHTg animals (Table 1). As an insulin sensitivity parameter, insulin-stimulated glycogenesis in skeletal muscles was significantly reduced in HHTg rats compared to Wistar controls, despite no differences in circulating adiponectin levels (Figure 1). Furthermore, the accumulation of ectopic hepatic lipids and lipotoxic intermediates was accompanied by increased oxidative stress parameters in the liver: both the GSH/GSSG ratio and activity of the antioxidant enzymes superoxide dismutase (SOD) and glutathioneperoxidase (GPx) were significantly decreased in untreated HHTg rats compared to the Wistar controls (Table 2). A marked accumulation of TAG in the liver was associated with changes in the gene expression of enzymes and transcriptional factors involved in hepatic lipid metabolism: relative mRNA expression of *Nrf2*, *Lpl,* and *Scd-1* were increased, while the gene expression of *Hmgcr* was decreased in untreated HHTg rats compared to Wistar controls (Figure 2). Hypertriglyceridemia was also associated with alterations in some hepatic proteins of the cytochrome P450 family (Figure 2). We observed increased mRNA gene expression of *Cyp4a*, *Cyp1a1*, *Cyp7a1*, and *Cyp2b1* in untreated HHTg animals. Hypertriglyceridemia was associated with markedly decreased serum levels of FGF21, a metabolic regulator with beneficial effects on glucose/lipid metabolism and insulin sensitivity (Figure 3). On the other hand, we observed no changes in circulating levels of fetuin-A.

### 2.2. Effect of Empagliflozin Treatment on Basic Metabolic Parameters and Insulin Sensitivity

As expected, urinary secretion of glucose reached extremely high levels after eight weeks of empagliflozin administration in both strains (Wistar controls: 13.97 ± 4.43 vs. 0.07 ± 0.01 mmol/g creatinine; prediabetic HHTg rats: 44.59 ± 1.74 vs. 0.15 ± 0.03 mmol/g creatinine). Empagliflozin treatment decreased glycemia and improved glucose tolerance, manifesting in significantly reduced serum fasting glucose, non-fasting glucose, and AUC in the empagliflozin-treated groups of rats (Table 1). Empagliflozin-treated rats exhibited increased insulin sensitivity in skeletal muscle, as measured by insulin-stimulated ^14^C-U-glucose into muscle glycogen (Figure 1). In Wistar controls, empagliflozin increased insulin sensitivity in visceral adipose tissue, as measured by insulin-stimulated lipogenesis. Significantly decreased levels of insulin also contributed to improved insulin sensitivity after empagliflozin administration (Table 1). However, empagliflozin administration did not affect circulating adiponectin or serum glucagon levels in either rat strain. 

### 2.3. Effect of Empagliflozin Treatment on Serum Lipids and Hepatic Lipid Metabolism

While empagliflozin-treated HHTg rats exhibited significantly decreased serum TAG concentrations, other serum lipids, including cholesterol, HDL-cholesterol, and NEFA, were not affected (Table 1). Ectopic TAG and lipotoxic diacylglycerols (DAG) accumulation in the liver reduced markedly after empagliflozin administration in controls and in the prediabetic group of rats (Table 2). Relative mRNA expression of the lipogenic enzymes *Fas* and *Scd1* and the lipogenic transcription factor *Srebf1* in the liver decreased significantly after empagliflozin treatment (Figure 2). On the other hand, the increased relative gene expression of *Hmgcr* in empagliflozin-treated HHTg rats was not accompanied by changes in hepatic cholesterol levels. In the liver, empagliflozin treatment significantly decreased the concentration of hepatic glycogen.

### 2.4. Effect of Empagliflozin Treatment on Hepatic Cytochrome P450 Family Proteins

Empagliflozin administration also affected some of the cytochrome P450 family proteins involved in lipid metabolism that can associate with the development and progression of fatty liver disease. As shown in Figure 2, the relative mRNA expression of *Cyp4a1*, *Cyp4a2*, *Cyp1a1*, *Cyp2b1,* and *Cyp7a1* were significantly decreased after empagliflozin treatment compared to untreated rats, whereas an elevation in gene expression of *Cyp2e1* was observed in the liver.

### 2.5. Effect of Empagliflozin Treatment on Hepatokines and Inflammatory and Oxidative Stress Parameters

**Figure 4:**
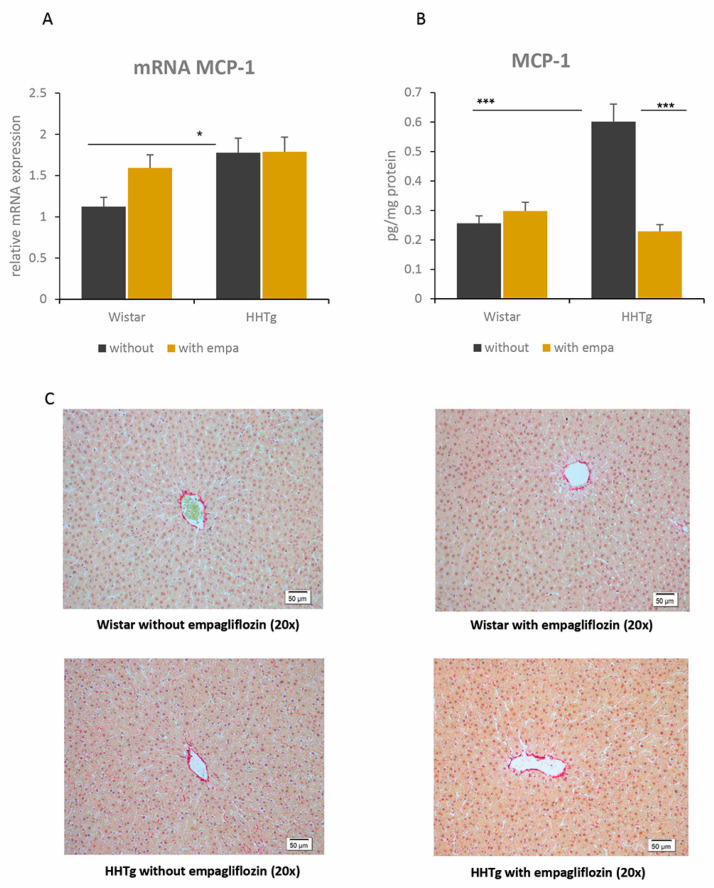
Effect of empagliflozin on inflammatory markers in the liver: relative gene expression (**A**) and concentration (**B**) of MCP-1 in Wistar control and prediabetic HHTg rats. (**C**)—Effect of empagliflozin on histological evaluation in prediabetic HHTg rats. Data are expressed as mean ± SEM; n = 8 for each group; * denotes *p* < 0.05, *** denotes *p* < 0.001.

Both of the circulating hepatokines monitored, FGF21 and fetuin-A, significantly decreased after empagliflozin administration in both strains (Figure 3). Serum levels of leptin and pro-inflammatory MCP-1 decreased in empagliflozin-treated animals (Table 1). In the liver, although there were no differences in the relative mRNA expression of *Mcp-1* or in histological evaluation, empagliflozin treatment markedly decreased hepatic MCP-1 concentrations in HHTg rats (Figure 4). 

In the liver, empagliflozin improved oxidative stress parameters but increased antioxidant enzyme activity of superoxide dismutase and glutathione peroxidase (Table 2) in empagliflozin-treated HHTg rats compared to untreated rats. However, empagliflozin did not affect the lipoperoxidation aldehydes MDA or 4-HNE. Alterations in hepatic oxidative stress parameters were associated with increased relative gene expression of transcriptional factor *Nrf2* (Figure 2).

## 3. Discussion

The current ‘multiple-hit’ theory of NAFLD pathogenesis posits a complex, synergistic process: the first hit involves lipid accumulation of hepatocytes [16] and the development of hepatic insulin resistance, while the second hit is characterized by liver damage and inflammation caused by oxidative stress. Favorably influencing lipid metabolism in the liver is essential for the mitigation of NAFLD, particularly in the early stages of development. Our study adds to the recent research investigating the role of empagliflozin; an SGLT-2 inhibitor recently reported to exert glycemic as well as pleiotropic effects on the progression of NAFLD and its symptoms. 

As expected, whole body weight reduced by 9% in association with a decreased relative weight of visceral adipose tissue. Our study excluded measurements of subcutaneous adipose tissue and lean body mass. However, studies using bioimpedance have shown that weight loss during empagliflozin-treatment likely contributes to a decrease in both visceral and subcutaneous adipose tissue in the absence of obvious change in lean body mass [17]. It has been suggested that this reduction in adipose tissue mass after empagliflozin-administration may be caused by energy loss due to increased glucose excretion and enhanced lipid mobilization [18]. In our study, body weight loss occurred despite a slight increase in food intake in empagliflozin-treated animals, a finding supported by other studies [12,19,20]. On the other hand, some studies report that increased ketone bodies act as a hunger signal to induce hyperphagia [21]. This mechanism is unlikely to have occurred in our study since the level of β-hydroxybutyrate in empagliflozin-treated animals remained unchanged. On the contrary, an increase in basal metabolism would contribute to weight loss via AMP/ATP regulation [19] or increased body temperature after empagliflozin treatment [22]. Although a modest reduction in body weight may partially contribute to the alleviation of NAFLD symptoms after empagliflozin administration, other mechanisms are likely to have more of an influence.

The reduction in glucose calories induced by empagliflozin is consistent with increased lipid mobilization, leading to reduced fat mass under the influence of hepatic lipid and glucose metabolism [18]. In the present study, empagliflozin treatment resulted in decreased neutral TAG as well as lipotoxic DAG concentration in the liver. The accumulation of intrahepatic lipid and lipotoxic intermediates induce insulin resistance and endoplasmic reticulum stress culminating in decreased hepatic function. Although there is a strong link between hepatic TAG accumulation and insulin resistance, TAG are neutral lipids responsible for generating lipotoxic intermediates, including DAG, ceramides, and lysophospholipids [23]. These lipotoxic compounds interfere with intracellular signaling pathways, causing further damage. DAG activates PKCε, which phosphorylates and inhibits the insulin receptor [24]. Decreased hepatic DAG is advanced as one of the mechanisms used by empagliflozin to improve insulin resistance in the liver as well in the peripheral tissues. However, markedly decreased insulinemia after empagliflozin administration can also improve insulin sensitivity. Several animal studies involving diabetic and obese rodents, although notable for their exclusion of lipotoxic intermediates, have observed decreased TAG in the liver after empagliflozin treatment [12,13]. In one human study involving diabetic subjects, empagliflozin treatment reduced liver fat content [25]. Intrahepatic accumulation of fatty acids induces insulin resistance and endoplasmic reticulum stress [26], two factors previously implicated in the intracellular mechanism of lipid accumulation and hepatic steatosis development.

In our study, although empagliflozin treatment modulated genes related to lipid synthesis (*Fas*, *Scd1*) and fatty acid metabolism (*Pparγ*), it had no effect on genes related to lipid oxidation or transport (*Pparα*, *Lpl*). Inhibition of de novo lipogenesis in the liver is understood to be an additional benefit of empagliflozin treatment, helping to reduce hepatic lipid accumulation. In our study, empagliflozin administration affected gene expression of the main lipogenic enzymes (*Scd1*, *Fas*) and transcription factors (*Srebp1*, *Pparγ*). Suppression of *Scd1* after empagliflozin was observed in another study with obese mice [11,12]. However, other animal studies of diabetic mice have produced conflicting results [27]. It is also possible that a decrease in SCD1 may be associated with CYP1A1. One study of CYP1A1^(−/−)^ mice demonstrated that elevated expression of *Cyp1a1* protects against NAFLD development, while dysregulation of the *Cyp1a1* gene is reportedly involved in the transport and metabolism of lipids [28,29]. Transcription factor SREBP1, one of the master regulators of hepatic lipogenesis, can aggravate hepatic steatosis [30]. Activation of PPARγ, another important factor involved in lipogenesis, may lead to increased lipid accumulation. Upregulation of both transcription factors is understood to increase lipid accumulation in the liver. Therefore, decreased gene expression of *Srebp1* and *Pparγ* plays a potential role in reducing hepatic lipid accumulation via suppression of lipogenesis, in turn contributing to improved hepatic insulin sensitivity. Reduced SREBP1 after empagliflozin treatment has also been demonstrated in studies of OLETF rats [19] and diet-induced obese mice [12].

Conversely, in our study, enzymes and transcription factors involved in lipid oxidation were not affected after empagliflozin. Although some animal studies have found a reduction in PPARα after empagliflozin administration, in our study, this transcription factor was unaffected. Interestingly, changes in PPARα have been observed in animal studies of diet-induced NAFLD [12].

In our study, gene expression of the cholesterol synthesis enzyme *Hmgcr* was partially affected. However, these changes did not affect hepatic cholesterol concentrations after empagliflozin, possibly in part due to the absence of alterations to SREBP2, the key transcription factor in cholesterol metabolism, following empagliflozin-treatment. 

Recent studies suggest that the transcription factor Nrf2 could play a role in lipid metabolism. It is understood that Nrf2 activation contributes to the beneficial effect of empagliflozin on hepatic lipid metabolism and lipid accumulation. In addition to improving oxidative stress, Nrf2 can affect and regulate lipid metabolism by inhibiting lipogenesis and influencing the gene expression of *Acc*, *Fas,* and *Hmgcr*, enzymes involved in lipid oxidation and synthesis [31]. In experimental models of NAFLD, Nrf2 has been shown to mediate crosstalk between lipid metabolism and antioxidant defense. Thus, induction of Nrf2 after empagliflozin could be a promising addition to NAFLD treatment.

Concerning its effect on plasma lipids, empagliflozin is understood to be activated as an indirect mechanism of glycosuria [32]. Furthermore, decreased hepatic glycogen content is strongly associated with empagliflozin-glycosuria promotion, with glucose loss occurring independently of insulin secretion. Hepatokines and lipids secreted from the liver control insulin sensitivity via both autocrine and paracrine signaling.

Changes in the gene expression of the enzyme superfamily cytochrome P450 (CYP) may also be involved in improving hepatic lipid metabolism following empagliflozin administration, thus alleviating symptoms of NAFLD. Some CYP enzymes are effective in the pathogenesis of NAFLD. Recent studies suggest that CYP2E1 plays an indispensable role in the development and progression of NAFLD [33], adapting its role during different phases. CYP2E1 maintains hepatic lipid homeostasis and, according to some animal studies, complements CYP4A enzymes during lipid utilization and oxidative stress, in particular, lipoperoxidation [34]. In the present study, increased mRNA expression in *Cyp2e1,* along with decreased expression of *Cyp4a1* and *Cyp4a2,* improved lipid accumulation following empagliflozin treatment. We can only assume that these effects are particularly effective during the early stages of NAFLD progression. Empagliflozin administration leads to glycogen utilization, lipid mobilization, and ketogenesis stimulation, serving to induce a pseudo-fasting state. Ketone bodies are known to prevent the degradation of CYP2E1 [35]. Therefore, upregulation of CYP2E1 is also associated with a starved state. Gene expression and CYP4A activity are markedly elevated in the liver tissues of NAFLD patients [36], as well as in diabetic and obese mice with hepatic steatosis [37]. Inhibiting CYP4A can ameliorate steatosis through reduced endoplasmic reticulum stress and improved insulin signaling. Thus, decreased CYP4A may assist in improving lipid metabolism and insulin resistance. Another cytochrome protein (CYP1A1), when overexpressed, may aggravate lipid peroxidation and oxidative stress [38]. Our results have shown that increases in CYP1A1 support the hypothesis of lipid peroxidation and development of NAFLD in HHTg rats.

Alterations in CYP enzymes after empagliflozin treatment can affect hepatic metabolites and oxidative stress products, thus modulating their pharmacotherapeutic effects. To the best of our knowledge, the effect of empagliflozin on cytochrome P450 proteins has yet to be investigated.

The beneficial effect of empagliflozin on NAFLD and its metabolic complications can be measured based on the levels of secreted hepatokines, which directly modulate glucose and lipid metabolism in the liver and establish crosstalk with adipose and muscle tissues. In the present study, empagliflozin administration markedly decreased circulating levels of fetuin-A in both strains. Surprisingly, however, it only slightly decreased levels of FGF21. Alterations in hepatokine secretion play a role in the pathogenesis of NAFLD and its complications [39]. Epidemiologic studies have shown that high serum levels of fetuin-A are independently associated with T2DM, insulin resistance, and metabolic syndrome. On the other hand, there are limited and conflicting data on the relationship between fetuin-A and NAFLD [40]. It has been suggested that fetuin-A acts as an important link between the liver, adipose tissue, and skeletal muscle [40]. The pro-inflammatory properties of fetuin-A exert a direct effect on the insulin receptor, thereby supporting insulin resistance [41]. It is reported that fetuin-A participates in low-grade inflammation by acting as an endogenous ligand for toll-like receptor 4 (TLR4), serving to promote a lipid-induced pro-inflammatory response [42]. The effect of empagliflozin on fetuin-A serum levels has not been established in either clinical or animal studies. However, in our study, we assume that decreased levels of fetuin-A after empagliflozin treatment are associated with improved insulin sensitivity and inflammation.

Compared to fetuin-A, FGF21 is a protective factor that regulates lipid metabolism and reduces lipid accumulation in hepatocytes through an insulin-independent pathway [43]. FGF21 also has a positive effect on insulin sensitivity and chronic inflammation [44]. One study found elevated serum levels of FGF21 in patients with NAFLD verified by magnetic resonance imaging [45]. FGF21 has also shown promise as a marker for NAFLD diagnosis, despite discrepant findings [44]. In the present study, serum levels of FGF21 decreased markedly in prediabetic rats compared to controls, supporting FGF21 as a suitable biomarker of NAFLD. Surprisingly though, empagliflozin treatment slightly decreased serum levels of FGF21 in our study. Data on how empagliflozin affects levels of circulating hepatokines are limited. Only one study with diet-induced obese mice with insulin resistance has been published thus far [22]. The authors reported that after prolonged empagliflozin treatment, serum levels and hepatic gene expression of *Fgf21* decreased in association with elevated fat utilization [22]. During empagliflozin administration, the effects of lipid oxidation and PPARα, as well as genetically determined dyslipidemia in HHTg rats, may explain the discrepancies between this study and ours. To fully elucidate the effects of empagliflozin on hepatokine secretion, further research is needed. Taken together, our results indicate that alterations in fetuin-A and FGF21 levels (after empagliflozin) relate more to improved insulin resistance and low-grade inflammation, whereas the effect on lipid metabolism is indirect and less extensive.

In the present study, empagliflozin treatment in prediabetic rats decreased pro-inflammatory MCP-1, attenuating the hepatic pro-inflammatory state. Decreased MCP-1 in the liver can help to reduce macrophage infiltration, leading to alleviation of hepatic steatosis and inflammation. In addition, MCP-1 regulates the expression of other pro-inflammatory cytokines, such as TNFα and IL-6. A recent study of diet-induced ApoE^−/−^ mice showed that empagliflozin decreases liver mRNA expression of *Mcp-1* [11]; however, in our study, the mRNA expression of *Mcp-1* was unchanged. In addition, we observed decreased MCP-1 concentration in the liver, while the ApoE^−/−^ mice study did not measure it. According to our results, decreased hepatic MCP-1 levels after empagliflozin treatment can be supported by improved oxidative stress and increased mRNA expression of *Nrf2*. Another study involving diet-induced obese mice found that the anti-inflammatory action of empagliflozin led to the suppression of *Nfκb* and *Tnfα* genes in the liver [12]. A variety of effects are potentially involved in alleviating inflammation following empagliflozin administration, such as reduced accumulation of lipotoxic intermediates, improved oxidative stress, and alterations in CYP proteins. Reduced MCP-1 may also be related to changes in hepatokine secretion, with fetuin-A acting as a strong link between metabolic dysregulation and inflammatory responses [43]. 

A crucial regulator of the cellular defense against oxidative stress is transcription factor Nrf2 [31]. Nrf2 also plays an important cytoprotective role in the development of NAFLD and has been evaluated as a potential target in the treatment and prevention of liver damage. A recent report showed that the regulation of the CYP450 enzyme is closely related to the Nrf2 pathway [46]. Thus, Nrf2 and some CYP enzymes may contribute to the process by which empagliflozin alleviates hepatic oxidative stress.

Empagliflozin regulates oxidative stress by stimulating antioxidant enzyme activity, SOD, and GPx via upregulation of Nrf2 rather than the direct inactivation of free radicals. In addition, increased activity of SOD and GPx may play a role in decreasing lipid peroxidation in the liver by participating in the removal of lipoperoxidation products. Increased activity of GPx after empagliflozin treatment was also linked to elevated glutathione levels and a suitable reduced/oxidized glutathione ratio. Glutathione is a sensitive marker of hepatic oxidative damage. However, in our study, we observed a trend for increased hepatic levels of glutathione following empagliflozin treatment. As compared to other experimental studies, empagliflozin suppresses hepatic oxidative stress via the activation of Nrf2/PPARγ crosstalk in mice, but it is about alcohol-induced liver damage [47]. 

Future research directions of experimental studies should be focused on investigating the circulating markers—hepatokines, adipocytokines, inflammatory parameters, or others, that would well reflect hepatic metabolic disorders in relation to the NAFLD development. A reliable parameter that would well reflect the stages of NAFLD development is still lacking in the human field of medicine.

## 4. Materials and Methods 

### 4.1. Animals and Diet

All experiments were performed in agreement with the Animal Protection Law of the Czech Republic (311/1997), which is in compliance with the European Community Council recommendations (86/609/ECC) on the use of laboratory animals and approved by the Ethics Committee of the Institute for Clinical and Experimental Medicine (53/2018, 21/6/2018). The study was performed on 6-month-old male Wistar rats (obtained from Charles River, Germany) as the control group and 6-month-old male HHTg rats (provided by the Institute for Clinical and Experimental Medicine, Prague, Czech Republic) as the non-obese prediabetic model. Rats were kept under temperature (22 °C) and humidity-controlled conditions under a 12/12 h light/dark cycle with free access to food (maintenance diet for rats and mice; Altromin, Lage, Germany) and drinking water. Wistar and HHTg rats were randomized into groups with or without empagliflozin treatment mixed as part of a standard diet at a dose of 10 mg/kg b.wt. for 8 weeks. 

At the end of the experiment, rats were sacrificed after light anesthesia (zoletil 5 mg/kg b.wt.) in a postprandial state. Aliquots of serum and tissue samples were collected and stored at −80 °C for further analysis. 

### 4.2. Analytic Methods, Biochemical Analysis

Serum levels of triacylglycerols (TAG), glucose, NEFA, ALT, and AST, as well as total and HDL cholesterol, were measured using commercially available kits (Erba Lachema, Brno, Czech Republic; Roche Diagnostics, Mannheim, Germany). 

Serum insulin, glucagon, leptin, and HMW adiponectin concentrations were determined using the Rat Insulin ELISA kit (Mercodia AB, Uppsala, Sweden) and the Rat Adiponectin ELISA kit (MyBioSource, San Diego, CA, USA). Serum MCP-1, TNFα, IL-6, and hsCRP were also measured using rat ELISA kits (BioSource International, San Diego, CA, USA; eBioscience-Bender MedSystems Biocenter, Austria; Alpha Diagnostics International, San Antonio, TX, USA, respectively). Serum β-hydroxybutyrate concentrations were measured using a colorimetric assay kit (Sigma-Aldrich, St. Louis, MO, USA). Serum levels of the hepatokines FGF21 and fetuin-A were determined by rat ELISA kits (MyBioSource, San Diego, CA, USA).

For the oral glucose tolerance test (OGTT), blood glucose was determined after a glucose load (300 mg/100g b.wt.) administered intragastrically after overnight fasting. Blood was drawn from the tail before the glucose load at 0 min and at 30, 60, and 120 min thereafter. 

For the determination of TAG and cholesterol in tissues, samples were extracted in chloroform/methanol. The resulting pellet was dissolved in isopropyl alcohol, with TAG content determined by enzymatic assay (Erba-Lachema, Brno, Czech Republic). To determine DAG in the liver, samples were extracted in dichloromethane/methanol. The resulting pellet was dissolved in isopropyl alcohol and isolated by thin-layer chromatography. The content of separated DAG was determined by enzymatic assay (Erba-Lachema, Brno, Czech Republic). As a marker of skeletal muscle insulin sensitivity, basal and insulin-stimulated glycogen synthesis was determined ex vivo in the isolated *musculus soleus* by measuring the incorporation of ^14^C-U glucose into glycogen as described previously [48]. 

For the determination of hepatic glycogen concentration, liver tissues were hydrolyzed in 30% potassium hydroxide (KOH) and precipitated by 96% ethanol overnight. The precipitate was then centrifuged, washed with ethanol, and dissolved in H_2_O. Aliquots were used to measure glycogen by glucose oxidase assay (Erba-Lachema, Brno, Czech Republic).

### 4.3. Oxidative Stress Parameters

The levels of reduced (GSH) and oxidized (GSSG) forms of glutathione were determined by the HPLC method with fluorescence detection using an HPLC diagnostic kit (Chromsystems, Gräfelfing, Germany). The activity of antioxidant enzymes superoxide dismutase and glutathione peroxidase were analyzed using Cayman Chemicals assay kits (Ann Arbor, MI, USA). MDA, a parameter of lipid peroxidation, was assessed by the HPLC method with fluorescence detection; 4-HNE, a sensitive product of lipoperoxidation, was determined using a rat ELISA kit (MyBiosource; San Diego, CA, USA).

### 4.4. Relative mRNA Expression

Total RNA was isolated from tissues using RNA Blue (Top-Bio, Vestec, Czech Republic). Reverse transcription and quantitative real-time PCR analysis were performed using the TaqMan RNA-to-CT 1-Step Kit, TaqMan Gene Expression Assays (Applied Biosystems, Waltham, MA USA), and the ViiA^TM^ 7 Real-Time PCR System (Applied Biosystems, Waltham, MA, USA). Relative expressions were determined after normalization against *β-actin* and *Hprt1* as an internal reference and calculated using the 2^−^^ΔΔCt^ method. Results were run in triplicate.

### 4.5. Histological Evaluation

Middle plane sagittal hepatic slices were fixed in formaldehyde for 48 h and processed using standard techniques into paraffin blocks. Sections were 3 μm thick and stained using routine hematoxylin and eosin staining and evaluated by a histopathologist.

### 4.6. Statistical Analysis

Two-way ANOVA was used to analyze the individual and combined effects of treatment and strain for treatment-vs.-strain interactions. All data analyzed were of normal distribution. Fisher’s LSD post-hoc test was used for variables showing evidence of treatment-vs.-strain interactions. The test was adjusted for multiple comparisons to determine whether empagliflozin treatment would significantly influence hepatic metabolic parameters in HHTg and Wistar strains. Statistical significance was set at a value of *p* < 0.05. All results are expressed as mean ± SEM. Statistical analysis was performed using StatSoft Statistica 14 software (StatSoft CZ; Prague, Czech Republic).

## 5. Conclusions

Our results show that empagliflozin treatment in prediabetic rats can mitigate hepatic steatosis by reducing hepatic triacylglycerols as well as lipotoxic intermediates. In the liver, empagliflozin modulated the expression of genes involved in lipogenesis and lipid storage, including *Scd1*, *Fas*, *Srebp1,* and *Pparγ*. However, the genes involved in lipid oxidation were not affected. We speculate that increased expression of the transcription factor Nrf2 contributed to the improvement of hepatic lipid metabolism and the alleviation of oxidative stress. Changes in the expression of the cytochrome P450 family proteins, in particular *Cyp2e1*, *Cyp1a1*, *Cyp4a1,* and *Cyp4a2*, which are involved in the pathogenesis of NAFLD, had a beneficial effect on lipid metabolism and insulin signaling after empagliflozin administration. In addition, markedly reduced levels of the important hepatokine fetuin-A were associated with improved insulin sensitivity and low-grade inflammation.

Our results highlight the therapeutic potential of empagliflozin in the treatment of NAFLD and its complications in the early phase of development independent of obesity and before the onset of diabetes. Thus, empagliflozin could ameliorate or delay NAFLD development in prediabetic patients; however, more clinical evidence is needed.

## Figures and Tables

**Table 1:** Body and serum metabolic characteristics.

	Wistar	Wistar + Empa	HHTg	HHTg + Empa	PS	PT	PI
Body weight (g)	568 ± 9	524 ± 14 **	465 ± 9	418 ± 9 **	<0.001	˂0.001	n.s.
Epididymal adipose tissue weight (mg/g)	1.75 ± 0.09	1.44 ± 0.10 **	1.66 ± 0.03	1.40 ± 0.05 *	n.s.	<0.001	n.s.
Fasting glucose (mmol/L)	5.64 ± 0.12	5.46 ± 0.14	6.86 ± 0.09	6.32 ± 0.16 **	<0.001	<0.05	n.s.
Non-fasting glucose (mmol/L)	6.74 ± 0.04	6.86 ± 0.13	8.68 ± 0.28	8.01 ± 0.18 *	˂0.001	n.s.	<0.05
Insulin (nmol/L)	0.252 ± 0.034	0.176 ± 0.016 *	0.299 ± 0.019	0.125 ± 0.014 ***	n.s.	<0.001	<0.05
Glucagon (pg/mL)	300.7 ± 40.4	267.6 ± 19.7	227.7 ± 5.2	240.3 ± 18.9	n.s.	n.s.	n.s.
AUC_0-180_ (mmol/L)	1303 ± 21	1227 ± 11 *	1604 ± 22	1507 ± 26 **	<0.001	<0.001	n.s.
Serum TAG (mmol/L)	1.20 ± 0.16	0.84 ± 0.12	4.86 ± 0.25	3.55 ± 0.26 ***	<0.001	<0.001	<0.05
Serum cholesterol (mmol/L)	2.03 ± 0.11	2.08 ± 0.13	2.05 ± 0.05	1.90 ± 0.06	n.s.	n.s.	n.s.
HDL-cholesterol (mmol/L)	0.97 ± 0.06	1.00 ± 0.09	0.94 ± 0.03	0.92 ± 0.02	n.s.	n.s.	n.s.
NEFA (mmol/L)	0.63 ± 0.04	0.64 ± 0.02	0.80 ± 0.05	0.79 ± 0.06	<0.01	n.s.	n.s.
HMW adiponectin (ng/mL)	1.10 ± 0.07	1.09 ± 0.08	1.00 ± 0.09	1.05 ± 0.04	n.s.	n.s.	n.s.
Leptin (ng/mL)	9.83 ± 0.93	6.23 ± 0.80 **	14.12 ± 0.91	7.39 ± 0.64 ***	<0.01	<0.001	n.s.
MCP-1 (pmol/L)	4.89 ± 0.20	4.30 ± 0.16 *	6.20 ± 0.18	5.50 ± 0.20 *	<0.001	<0.01	n.s.
TNFα (pg/mL)	2.33 ± 0.24	2.36 ± 0.26	3.13 ± 0.23	3.33 ± 0.31	<0.01	n.s.	n.s.
IL-6 (pg/mL)	91.96 ± 4.82	83.32 ± 4.88	114.04 ± 5.87	126.74 ± 6.93	<0.001	n.s.	n.s.
hsCRP (μg/mL)	1.94 ± 0.22	1.60 ± 0.18	1.49 ± 0.09	1.74 ± 0.16	n.s.	n.s.	n.s.
β-hydroxybutyrate (μmol/L)	1.00 ± 0.12	0.83 ± 0.09	1.13 ± 0.09	1.11 ± 0.09	<0.05	n.s.	n.s.

Two-way ANOVA results: PS denotes the significance of Wistar vs. HHTg (strain effects), PT denotes the significance of empagliflozin (treatment effects); PI denotes the significance of empagliflozin in both strains (treatment vs. strain interaction). For multiple comparisons Fisher’s LSD post-hoc test was used; * denotes *p* ˂ 0.05; ** denotes *p* ˂ 0.01; *** denotes *p* ˂ 0.001. Data are mean ± SEM; n = 8 for each group. HHTg—hereditary hypertriglyceridemic rats; AUC—area under curve; TAG—triacylglycerols; MCP-1—monocyte chemoattractant protein; TNFα—tumour necrosis factor α; IL-6—interleukin-6.

**Table 2:** Hepatic lipid and oxidative stress parameters.

	Wistar	Wistar + Empa	HHTg	HHTg + Empa	PS	PT	PI
Relative liver weight (mg/g)	2.42 ± 0.06	2.37 ± 0.05	3.15 ± 0.08	3.11 ± 0.06	˂0.001	n.s.	n.s.
TAG in the liver (μmol/g)	8.21 ± 0.39	7.71 ± 0.36	11.79 ± 0.44	9.67 ± 0.29 ***	<0.001	<0.01	<0.05
DAG in the liver (μmol/g)	1.85 ± 0.07	1.30 ± 0.09 ***	2.27 ± 0.12	1.39 ± 0.07 ***	<0.01	˂0.001	n.s.
Cholesterol in the liver (μmol/g)	12.04 ± 0.59	12.29 ± 0.56	13.68 ± 0.36	14.20 ± 0.35	<0.001	n.s.	n.s.
Glycogen in the liver (μmol/g)	188.75 ± 20.08	84.12 ± 8.67 ***	315.68 ± 24.03	223.14 ± 14.89 ***	<0.001	<0.001	n.s.
GSH/GSSG (μmol/g)	23.17 ± 1.81	24.57 ± 3.25	16.39 ± 0.87	22.89 ± 1.25 *	<0.05	n.s.	n.s.
SOD (U/mg)	0.103 ± 0.006	0.103 ± 0.016	0.093 ± 0.009	0.173 ± 0.016 ***	<0.05	<0.001	<0.001
GPx (μM NADPH/min/mg)	224.9 ± 22.7	275.6 ± 33.1	149.3 ± 18.5	234.5 ± 16.3 *	<0.05	<0.01	n.s.
MDA (nmol/mg)	3.82 ± 0.38	3.47 ± 0.46	3.92 ± 0.37	3.38 ± 0.72	n.s.	n.s.	n.s.
4-HNE (nmol/mg)	0.61 ± 0.05	0.63 ± 0.04	0.53 ± 0.10	0.52 ± 0.03	n.s.	n.s.	n.s.
Serum ALT (μkat/L)	1.05 ± 0.09	1.25 ± 0.17	1.28 ± 0.10	1.36 ± 0.13	n.s.	n.s.	n.s.
Serum AST (μkat/L)	2.62 ± 0.11	2.76 ± 0.15	3.34 ± 0.21	3.25 ± 0.14	˂0.01	n.s.	n.s.

Two-way ANOVA results: PS denotes the significance of Wistar vs. HHTg (strain effects), PT denotes the significance of empagliflozin (treatment effects); PI denotes the significance of empagliflozin in both strains (treatment vs. strain interaction). For multiple comparisons, Fisher’s LSD post-hoc test was used; * denotes *p* ˂ 0.05; *** denotes *p* ˂ 0.001. Data are mean ± SEM; n = 8 for each group. HHTg—hereditary hypertriglyceridemic rats; TAG—triacylglycerols; DAG—diacylglycerols; GSH—glutathione; GSSG—oxidized form of glutathione; SOD—superoxide dismutase; GPx—glutathioneperoxidase; MDA—malondialdehyde; 4-HNE—4-hydroxynonenal.

## Data Availability

All datasets generated for this study are included in the article.

## Abbreviations

ACC	acetyl-coenzyme A carboxylase
AMPK	adenosine monophosphate-activated protein kinase
CYP450	cytochrome P450
DAG	diacylglyceroles
FAS	fatty acid synthase
FGF21	fibroblast growth factor 21
GSH	reduced form of glutathione
GSSG	oxidized form of glutathione
HMGCR	3-hydroxy-3-methylglutaryl-coenzyme A reductase
4-HNE	4-hydroxynonenale
hsCRP	high-sensitivity C-reactive protein
IL-6	interleukin 6
MDA	malondialdehyde
MAPK	mitogen-activated protein kinase
MCP-1	monocyte chemoattractant protein-1
NAFLD	non-alcoholic fatty liver disease
NEFA	non-esterified fatty acid
NF-κB	nuclear factor kappa B
NRF2	nuclear factor erythroid-2-related factor 2
PPARα	peroxisome proliferator-activated receptor alpha
PPARγ	peroxisome proliferator-activated receptor gamma
SCD	stearoyl-coenzyme A desaturase
SREBP1	sterol regulatory element-binding protein 1
TAG	triacylglycerols
TNFα	tumor necrosis factor alpha
